# COVID-19 and CAP: What Changed in Training and Practice for Early Career Child/Adolescent Psychiatrists?

**DOI:** 10.1192/j.eurpsy.2022.203

**Published:** 2022-09-01

**Authors:** A. Seker

**Affiliations:** Cambridge and Peterborough NHS Foundation Trust, Child/adolescent Psychiatry, Cambridge, United Kingdom

## Abstract

Covid-19 pandemic has affected early career psychiatrists (ECP) and psychiatric trainees significantly. Child and Adolescent Psychiatry (CAP) specialists and trainees have a particular position in the pandemic as redeployments to adult units are possibly more disorientating for these psychiatrists normally working in paediatric settings. Redeployments and abrupt but potentially permanent changes to the delivery of service and training are explored via the Covid-19 and Early Career Psychiatrists survey which was prepared and disseminated by the EPA ECP Committee and the European Federation of Psychiatric Trainees (EFPT). The CAP related data from the survey will be discussed in this presentation and the implications on the future of CAP will be considered with particular emphasis on training.

**Disclosure:**

No significant relationships.

